# Inpatient rehabilitation outcomes in acquired brain injury: a retrospective study

**DOI:** 10.1590/1806-9282.20260575

**Published:** 2026-09-28

**Authors:** Sefa Gümrük Aslan, Şehri Bariş Saklica, Sinem Uyar Köylü, Kurtuluş Köklü

**Affiliations:** 1 University of Health Sciences, Ankara Gaziler Physical Medicine and Rehabilitation Health Application and Research Center, Physical Medicine and Rehabilitation – Ankara, Türkiye.

**Keywords:** Brain injuries, Brain injuries, traumatic, Neurological rehabilitation, Treatment outcome, Recovery of function, Glasgow Coma Scale

## Abstract

**OBJECTIVE:**

The objective of this study was to describe the demographic, clinical, cognitive, and functional characteristics of acquired brain injury patients, analyze complications, and evaluate inpatient rehabilitation outcomes.

**METHODS:**

Retrospective review of 50 acquired brain injury patients undergoing multidisciplinary inpatient rehabilitation (2019–2024). Glasgow Coma Scale, post-traumatic amnesia, cognitive scales (Mini-Mental State Examination, Montreal Cognitive Assessment, Rancho Los Amigos), Functional Ambulation Scale, and complications were assessed.

**RESULTS:**

Traumatic etiology comprised 76% of cases; 72% were severe (Glasgow Coma Scale 3–8). Prolonged post-traumatic amnesia (>24 h) was significantly associated with severe injury (p<0.001). Spasticity (64%), contractures (42%), speech disorders (42%), and dysphagia (34%) were most prevalent. Functional Ambulation Scale score 0 decreased from 58 to 42% post-rehabilitation; Functional Ambulation Scale 3–5 increased from 24 to 46% (Wilcoxon signed-rank test, p<0.001).

**CONCLUSION:**

Structured multidisciplinary rehabilitation was associated with measurable functional gains in acquired brain injury patients despite severe deficits and frequent complications; however, the absence of a control group precludes causal conclusions.

## INTRODUCTION

Acquired brain injury (ABI) encompasses damage to the central nervous system from both traumatic and non-traumatic etiologies, resulting in physical, cognitive, and psychosocial impairments. Traumatic brain injury (TBI) is a leading global health burden with an annual incidence of 150–300 per 100,000; males are affected two to three times more frequently, particularly those aged 18–25 years^1–5^. Non-traumatic ABI includes focal lesions, anoxic injury, tumors, aneurysms, and CNS infections^6^. ABI patients face a high risk of secondary complications-spasticity, contractures, post-traumatic epilepsy, dysphagia, and heterotopic ossification-that impair recovery^7–9^. Early, multidisciplinary rehabilitation reduces these complications, enhances neuroplasticity, and improves long-term outcomes^10–13^. This study aimed to describe the demographic, clinical, cognitive, and functional profile of ABI patients admitted to a specialized inpatient rehabilitation center and evaluate outcomes stratified by injury severity.

## METHODS

### Study design and participants

This retrospective, single-center study reviewed records of 350 ABI patients hospitalized for inpatient rehabilitation between 2019 and 2024. Inclusion criteria were as follows: age ≥18 years, confirmed ABI diagnosis, absence of comorbidities affecting cognitive or functional status, and complete medical records. Of the 350 patients screened, 300 were excluded: 128 due to missing admission and/or discharge assessment data (e.g., baseline or discharge GCS, FAS, or cognitive scale scores not recorded), 130 due to other incomplete chart/file data required for this analysis, and 42 because the patient was discharged prematurely for an acute medical emergency before completing the rehabilitation program and therefore lacked discharge outcome data. After screening, 50 patients with complete admission-to-discharge records were included. The study was approved by the Ethics Committee of Gaziler Physical Medicine and Rehabilitation Training and Research Hospital (Date: 26.10.2023; Protocol No: 29/008).

### Data collection

Demographic variables, date of injury, initial GCS score, PTA duration, and injury etiology were recorded. PTA duration was categorized as none, <1 h, 1–24 h, or >24 h based on retrospective chart documentation; as intermediate durations were rarely specified in the medical records, all patients fell into either the none or >24-h categories. Muscle strength was evaluated using the MRC scale. Cognitive status was assessed with Mini-Mental State Examination (MMSE), Montreal Cognitive Assessment (MoCA), and the Rancho Los Amigos Scale. Functional mobility was evaluated with FAS.

### Rehabilitation protocol

All patients received a standardized neurological rehabilitation program, delivered 5 days per week for 45 min per session, regardless of severity group. The program consisted of neurophysiological exercises, balance exercises, postural training exercises, proprioceptive exercises, and ambulation and transfer exercises, in addition to ROM exercises, verticalization training, speech-cognitive therapy, ADL exercises, and hand therapy, provided under the supervision of physiatrists, physiotherapists, occupational therapists, and speech-language pathologists.

### Statistical analysis

Statistical analyses were performed using SPSS v27.0. Categorical variables were expressed as frequencies and percentages; continuous variables as means±SD. Chi-square tests were applied for categorical comparisons. Student's t-test or Mann-Whitney U test was used for continuous variables; one-way ANOVA for three-group comparisons. Normality was assessed using the Kolmogorov-Smirnov test. p<0.05 was considered statistically significant. Given the disparate subgroup sizes (severe n=36, moderate n=8, mild n=6), post-hoc power analysis was performed for the one-way ANOVA comparisons of cognitive outcomes using the observed effect sizes, group sizes, and an alpha of 0.05. Kolmogorov-Smirnov/Shapiro-Wilk testing indicated that MMSE and Rancho Los Amigos scores were significantly non-normally distributed in the severe subgroup (and, for Rancho Los Amigos, also in the mild subgroup), which is noted as a caveat on the corresponding ANOVA results given the small and unequal subgroup sizes. Within-group pre-post change in FAS score (admission vs. discharge) was assessed using the Wilcoxon signed-rank test given its ordinal, paired nature. An independent-samples t-test was used to compare traumatic and non-traumatic etiology groups on muscle strength and cognitive outcomes, and chi-square tests were used to compare these groups on complication frequency and FAS scores. The study was reported in accordance with the STROBE statement.

## RESULTS

### Demographic characteristics

Fifty patients were included (mean age 34.68±12.88 years; 80% male). Mean BMI was 25.22±3.83 kg/m^2^. High school graduates comprised 62%; 52% were single.

### Clinical characteristics

Mean time since injury was 15.62±14.08 months. Traumatic etiology accounted for 76% of cases. Mean GCS was 6.5±4.17; 72% were classified as severe (GCS 3–8), 16% moderate, and 12% mild. Chi-square analysis confirmed a significant association between severity and prolonged PTA (p<0.001): 83.3% of severe TBI patients experienced PTA >24 h vs. 25% in the moderate and 0% in the mild group (Table 1). Mean MMSE was 23.2±4.60, MoCA 19.22±5.53, and Rancho Los Amigos 6.94±1.67.

**Table 1:** Clinical characteristics of patients with acquired brain injury (n=50).

Variable		Value (n=50)
Time since injury (months)		15.62±14.08
Etiology, n (%)		
	Traumatic	38 (76)
	Non-traumatic	12 (24)
	Glasgow Coma Scale (GCS)	6.5±4.17
GCS classification, n (%)		
	Severe (3–8)	36 (72)
	Moderate (9–12)	8 (16)
	Mild (13–15)	6 (12)
Post-traumatic amnesia, n (%)		
	None	18 (36)
	<1 h	0
	1–24 h	0
	>24 h	32 (64)
	Upper extremity muscle strength	3.46±1.21
	Lower extremity muscle strength	3.42±1.07
	MMSE	23.2±4.60
	MoCA	19.22±5.53
	Rancho Los Amigos Scale	6.94±1.67

Continuous variables are presented as mean±standard deviation (SD). Categorical variables are expressed as frequency and percentage (n, %). MMSE: Mini-Mental State Examination; MoCA: Montreal Cognitive Assessment; GCS: Glasgow Coma Scale.

### Complications

Spasticity was the most prevalent complication (64%), followed by joint contractures and speech disorders (42% each), dysphagia (34%), post-traumatic epilepsy (24%), neurogenic bladder (18%), and neurogenic bowel dysfunction (12%). Contracture was significantly more frequent in the severe TBI group (p=0.046).

### Functional and cognitive outcomes

Cognitive assessments showed no statistically significant differences between severity groups for MMSE, MoCA, or Rancho Los Amigos scores (all p>0.05; Table 2). Post-hoc power analysis indicated that the study had limited power to detect between-group differences of the magnitude observed (achieved power: 11.8% for MMSE, 24.1% for MoCA, 43.9% for Rancho Los Amigos, at α=0.05), so these non-significant findings should be interpreted with caution given the substantial risk of Type II error rather than as evidence of true equivalence between groups. Descriptively, FAS scores shifted post-rehabilitation: FAS 0 decreased from 58 to 42%, while combined FAS 3–5 increased from 24 to 46% (Table 3); the p-values reported in Table 3 reflect between-group (severity) comparisons at each timepoint rather than within-group pre-post comparisons. Within-group pre-post change was formally tested with a Wilcoxon signed-rank test, which confirmed a statistically significant improvement in FAS from admission to discharge (21 patients improved, 0 worsened, 29 unchanged; Z=-4.078, p<0.001). In the severe group, FAS 0 decreased from 58.2 to 47.4%, and FAS 3–5 rose from 25 to 44.2%. In the moderate group, 50% reached FAS 3 post-treatment; in the mild group, FAS 0 decreased from 83.4 to 33%. To assess whether pooling traumatic and non-traumatic etiologies affected the cognitive and muscle strength outcomes, an independent-samples t-test compared traumatic (n=38) and non-traumatic (n=12) patients on upper extremity strength, lower extremity strength, MMSE, MoCA, and Rancho Los Amigos scores. No significant differences were found between etiology groups on any measure [upper extremity strength: t(48)=0.410, p=0.683; lower extremity strength: t(48)=-0.294, p=0.770; MMSE: t(48)=0.456, p=0.650; MoCA: t(48)=0.097, p=0.923; Rancho Los Amigos: t(48)=-0.734, p=0.467], with small effect sizes throughout (Cohen's d range 0.03–0.24), suggesting that analyzing traumatic and non-traumatic ABI together did not materially affect these outcome comparisons. Chi-square tests comparing traumatic and non-traumatic patients on complication frequency and functional status likewise showed no significant differences: spasticity [χ^2^(1)=0.049, p=0.825], contracture [χ^2^(1)=0.001, p=0.979], neurogenic bladder [χ^2^(1)=0.524, p=0.469], neurogenic bowel [χ^2^(1)=2.527, p=0.112], admission FAS [χ^2^(4)=1.958, p=0.743], and discharge FAS [χ^2^(5)=4.574, p=0.470].

**Table 2:** Comparison of clinical variables between brain injury severity groups.

Variable	Severe TBI (n=36, 72%)	Moderate TBI (n=8, 16%)	Mild TBI (n=6, 12%)
Post-traumatic amnesia, n (%)			p-value
None	6 (16.7)	6 (75.0)	6 (100) <0.001
<1 h	0	0	0
1–24 h	0	0	0
>24 h	30 (83.3)	2 (25.0)	0
MMSE	22.9±4.9	24.6±3.7	23.3±3.8 0.627
Rancho Los Amigos Scale	6.7±1.5	7.8±2.3	7.7±0.5 0.123
MoCA	18.5±6.1	21.6±3.5	20.3±3.4 0.312

Values are presented as mean±SD for continuous variables and as frequency and percentage (n, %) for categorical variables. Categorical variables were compared using chi-square test; continuous variables using one-way ANOVA. MMSE: Mini-Mental State Examination; MoCA: Montreal Cognitive Assessment; TBI: traumatic brain injury. p<0.05 was considered statistically significant.

**Table 3:** Comparison of Functional Ambulation Scale scores before and after treatment across brain injury severity groups.

FAS score	Group 1 GCS 3–8 (n=36)	Group 2 GCS 9–12 (n=8)	Group 3 GCS 13–15 (n=6)
Before treatment			p-value
0	21 (58.2)	3 (37.5)	5 (83.4) 0.060
1	5 (13.8)	0 (0)	1 (16.6)
2	1 (2.0)	2 (25.0)	0 (0)
3	9 (25.0)	2 (25.0)	0 (0)
4	0 (0)	1 (12.5)	0 (0)
After treatment			p-value
0	17 (47.4)	2 (25.0)	2 (33.0) 0.277
1	2 (5.5)	0 (0)	0 (0)
2	1 (2.7)	1 (12.5)	2 (33.0)
3	11 (30.5)	4 (50.0)	1 (17.0)
4	4 (11.0)	0 (0)	0 (0)
5	1 (2.7)	1 (12.5)	1 (17.0)

Values are presented as frequency and percentage (n, %). FAS scores range from 0 (non-ambulatory) to 5 (normal ambulation). The p-values shown reflect chi-square comparisons across the three severity groups at each timepoint (before and after treatment, respectively); within-group pre-post change (admission vs. discharge, whole cohort) was tested separately using the Wilcoxon signed-rank test (Z=-4.078, p<0.001; 21 patients improved, 0 worsened, 29 unchanged). GCS: Glasgow Coma Scale; FAS: Functional Ambulation Scale.

## DISCUSSION

Traumatic brain injury (TBI) continues to be a significant public health concern due to its high potential for long-term disability and the complex range of physical, cognitive, and psychosocial impairments it may cause. This study offers a comprehensive evaluation of clinical, functional, and cognitive characteristics in patients with acquired brain injury, classified according to initial injury severity. One of the key strengths of the present research lies in its multidimensional approach, incorporating not only demographic and injury-related variables but also detailed assessments of complications, cognitive functioning, and post-treatment functional outcomes. By stratifying patients into severity-based subgroups and conducting within-group and between-group analyses, the study provides valuable insights into how injury severity correlates with outcomes such as post-traumatic amnesia, spasticity, contracture, and frontal lobe functioning. The longitudinal comparison of FAS scores before and after treatment underscores the dynamic nature of recovery, revealing a notable shift toward higher FAS scores in all groups, highlighting the importance of structured follow-up and tailored rehabilitation strategies even for patients with mild to moderate presentations. The findings indicate that the majority of patients with severe TBI experienced higher rates of complications such as post-traumatic epilepsy, spasticity, and joint contractures, consistent with prior literature. Post-traumatic amnesia lasting more than 24 h was most prevalent in the severe TBI group, whereas no cases of PTA were observed in mild TBI. Dysphagia was also notable in moderate and severe cases. Interestingly, deep vein thrombosis (DVT) was reported only in the mild group, which may reflect coincidental findings or limitations in documentation. In the comparison of cognitive assessments among patients with varying severity of brain injury, no statistically significant differences were found in terms of MMSE, Rancho Los Amigos Scale, or MoCA scores. Although the severe group exhibited lower mean scores in all three evaluations, these differences did not reach statistical significance. This suggests a trend toward more impaired cognitive and behavioral functioning in patients with more severe injury, but the small sample size and variability within groups may have limited the ability to detect significant effects. Our results align with recent studies emphasizing the importance of multidisciplinary rehabilitation approaches in TBI. For example, Varuges et al. demonstrated statistically significant improvements in walking distance after inpatient rehabilitation in a similar population^14^. In this study, a high prevalence of neuromuscular and neurological complications was observed. Spasticity emerged as the most common complication, affecting 64% of patients, aligning with previous reports suggesting that up to two-thirds of individuals with moderate to severe brain injury experience spasticity during recovery^15^. Joint contractures and speech disorders were also notably frequent, underscoring the need for early and sustained physiotherapeutic and speech-language interventions^16^. Dysphagia, seen in 34% of cases, is a well-documented consequence of TBI associated with increased risk of aspiration pneumonia and prolonged hospital stays^17^. Post-traumatic epilepsy affected 24% of patients, consistent with prior findings reporting an incidence of 10–25% in moderate to severe TBI^18^. Neurogenic bladder and bowel dysfunction were present in 18 and 12% of patients, respectively, reflecting common autonomic disturbances that accompany more extensive brain injuries^19^. Rare but clinically significant complications such as hydrocephalus and DVT were each identified in 2%. These findings emphasize the necessity of comprehensive, multidisciplinary rehabilitation programs capable of addressing a wide spectrum of secondary complications. It is also important to consider that spasticity and joint contractures may limit functional gains despite cognitive recovery, underscoring the need for early spasticity management and joint protection strategies. Previous studies have consistently demonstrated that PTA duration is a strong indicator of TBI severity and a reliable predictor of long-term cognitive and functional recovery. Ponsford et al.^20^ reported that longer PTA is significantly associated with poorer neuropsychological outcomes. Similarly, Walker et al.^21^ demonstrated that PTA duration was the most important injury-related predictor of long-term global functional outcome^22^. In the present study, 83.3% of severe TBI patients experienced PTA longer than 24 h, compared to 25% in the moderate and 0% in the mild groups, reinforcing the utility of PTA as a marker of injury severity. Despite this significant difference, our findings did not show statistically significant between-group differences in cognitive and functional outcomes. These results may be attributed to early timing of assessments, limited subgroup sizes, and the homogenizing effect of a standardized, intensive inpatient rehabilitation program. Prior research has indicated that cognitive improvements during the inpatient phase may be similar across severity groups when structured multidisciplinary rehabilitation is provided^23^. Andelic et al.^12^ reported a high prevalence of post-traumatic complications even 10 years post-injury, including spasticity, joint contractures, and neurogenic dysfunctions. Similarly, Ponsford et al.^20^ found that long-term outcomes are often influenced by initial injury severity, but variability in individual recovery trajectories remains significant. The presence of dysphagia and speech disorders across all TBI groups underscores the importance of comprehensive interdisciplinary management regardless of injury severity. The significantly higher incidence of joint contractures in the severe TBI group highlights the need for proactive contracture prevention strategies during inpatient rehabilitation. The present study evaluated the impact of inpatient rehabilitation on ambulation in patients stratified by GCS. While all groups exhibited improvements in FAS scores post-treatment, these did not reach statistical significance. Al-Jarrah et al.^24^ found that patients with severe and moderate TBI showed significant improvements in FIM scores following rehabilitation, whereas those with mild TBI did not, suggesting that injury severity may influence the degree of functional recovery achievable. Bilgin et al.^25^ reported that GCS scores correlated with initial functional status but were not strong predictors of discharge outcomes, underscoring the importance of comprehensive assessments and individualized rehabilitation plans. Future studies with larger cohorts and longer follow-up periods are necessary to elucidate the full impact of rehabilitation on ambulation and other functional domains. This study offers several strengths. The categorization of patients based on GCS scores into severity subgroups enabled detailed analysis of injury severity–outcome relationships. The inclusion of multiple standardized scales-FAS, MMSE, MoCA, and Rancho Los Amigos-allowed for multidimensional assessment of cognitive and motor functions. Conducting the study in a specialized neurorehabilitation center ensured consistency in treatment protocols, strengthening internal validity. However, its retrospective and single-center design limits generalizability and introduces potential bias. Of the 350 patients initially screened, only 50 (14.3%) met inclusion criteria (128 excluded for missing admission/discharge assessment data, 130 for other incomplete chart data, and 42 for premature discharge due to an acute medical emergency); systematic differences between included and excluded patients-particularly the possibility that those discharged early for medical instability differed in injury severity or complication burden from those completing the program-cannot be ruled out and may limit the representativeness of the study sample. A formal comparison of included versus excluded patients on baseline demographic or clinical variables was not feasible, since the majority of exclusions were themselves due to missing or incomplete chart data for these same variables. The small subgroup sizes substantially reduced statistical power, as confirmed by post-hoc power analysis (achieved power ranging from 11.8 to 43.9% across cognitive outcomes), such that the non-significant between-group findings should be regarded as inconclusive rather than as evidence of no difference. Traumatic and non-traumatic etiologies were analyzed together given the limited sample size (n=38 vs. n=12); post-hoc comparisons found no significant differences between these groups on muscle strength, MMSE, MoCA, or Rancho Los Amigos scores, nor on complication frequency (spasticity, contracture, neurogenic bladder or bowel) or admission/discharge FAS distribution (all p>0.05, small effect sizes), suggesting this pooling did not materially affect the reported outcomes, though the small non-traumatic subgroup limits the power to detect subtler etiology-specific patterns. The pre-post change in FAS scores was formally tested with a Wilcoxon signed-rank test (Z=-4.078, p<0.001), confirming a statistically significant improvement; however, this whole-cohort result was not stratified by severity subgroup given the small moderate (n=8) and mild (n=6) group sizes, which limits inference about whether the improvement was uniform across severity levels. Additionally, assessments were limited to the inpatient period, precluding evaluation of long-term recovery trajectories, and the absence of a non-rehabilitation or alternative-treatment comparison group means that observed functional improvements cannot be firmly attributed to the intervention rather than to spontaneous recovery. Although session frequency and duration (5 days/week, 45 min/session) were standardized across all patients, length of stay varied considerably (14–98 days), so total treatment dose was not constant and was not analyzed as a potential confounder. Future prospective, multicenter, controlled studies with larger populations, a priori power calculations, and long-term follow-up are needed.

## CONCLUSION

This study provides valuable insights into the clinical characteristics, complications, and functional outcomes of ABI patients across varying injury severity levels. The findings underscore the importance of early, multidisciplinary rehabilitation and individualized treatment plans. Although statistical significance was not observed in all outcomes, meaningful shifts in functional status were observed post-rehabilitation. Future prospective studies with larger samples and longer follow-up are needed to elucidate long-term recovery patterns in this population.

## Data Availability

The datasets generated and/or analyzed during the current study are available from the corresponding author upon reasonable request.
